# Percutaneous intramyocardial septal radiofrequency ablation: a novel treatment for drug-refractory non-obstructive hypertrophic cardiomyopathy with severe septal hypertrophy

**DOI:** 10.1136/heartjnl-2024-325334

**Published:** 2025-03-07

**Authors:** Huiyi Wang, Jing Li, David H Hsi, Wenxia Li, Shengjun Ta, Yiyu Jiao, Bo Shan, Lingxiao Chang, Xumei Ou, Lu Yao, Bo Wang, Jing Wang, Changhui Lei, Liwen Liu

**Affiliations:** 1 Department of Ultrasound, Xijing Hospital of Air Force Military Medical University, Xian, Shaanxi, China; 2 Department of Cardiology, Stafford Hospital, Stafford, Virginia, USA

**Keywords:** Echocardiography, Cardiomyopathy, Hypertrophic, Treatment Outcome

## Abstract

**ABSTRACT:**

**Background:**

Patients with drug-refractory non-obstructive hypertrophic cardiomyopathy (NOHCM) lack effective invasive treatment options. This study aimed to evaluate the safety and effectiveness of percutaneous intramyocardial septal radiofrequency ablation (PIMSRA, Liwen procedure) in patients with NOHCM and severe septal hypertrophy (≥28 mm).

**Method:**

This single-arm, open-label, prospective study enrolled 20 adult patients with drug-refractory NOHCM between June 2017 and June 2023. Patients underwent PIMSRA and were followed for a median of 15 months. Outcomes included changes in septal thickness, quality of life (Kansas City Cardiomyopathy Questionnaire-12, KCCQ-12) and myocardial function.

**Results:**

No major adverse clinical events occurred within 30 days after the procedure. The maximum interventricular septal thickness decreased significantly from 31.3 mm to 17.4 mm (mean difference: −13.9 mm; 95% CI −15.92 to −11.88). Left atrial volume index and left ventricular mass index also decreased significantly. Improvements in global longitudinal strain and global radial strain were observed, indicating possibly enhanced myocardial performance. KCCQ-12 scores improved from 65.6 to 84.4 (mean difference: 18.78; 95% CI 11.62 to 25.93). Patients after PIMSRA were not found to have an increased risk of arrhythmias such as atrial fibrillation, high-grade conduction block or non-sustained ventricular tachycardia during the follow-up.

**Conclusions:**

PIMSRA was associated with a reduction of myocardial septal thickness and improvement in functional status in patients with NOHCM. The absence of major adverse events is also encouraging, but larger studies with a control arm are needed to confirm long-term safety.

**Trial registration number:**

ChiCTR1900020530.

**WHAT IS ALREADY KNOWN ON THIS TOPIC**

- Patients with drug-refractory non-obstructive hypertrophic cardiomyopathy (NOHCM) have limited invasive treatment options, often leading to poor quality of life and progression to advanced heart failure.
- Current guidelines recommend medical therapy as the primary treatment for NOHCM, but a subset of patients remain symptomatic despite optimal medical management.
- Surgical myectomy or heart transplantation are considered for severe cases, but these options carry significant risks and are not suitable for all patients.

**WHAT THIS STUDY ADDS**

- Percutaneous intramyocardial septal radiofrequency ablation (PIMSRA) resulted in a significant reduction of interventricular septal thickness (mean reduction: 13.9 mm) in patients with NOHCM and severe septal hypertrophy (≥28 mm).
- PIMSRA is associated with improvements in quality of life, as measured by the Kansas City Cardiomyopathy Questionnaire-12, and enhancements in myocardial mechanics.
- The procedure was not associated with major adverse clinical events within 30 days, suggesting a potentially favourable safety profile in this patient population.

**HOW THIS STUDY MIGHT AFFECT RESEARCH, PRACTICE OR POLICY**

- PIMSRA may offer a new, minimally invasive treatment option for patients with drug-refractory NOHCM and severe septal hypertrophy, potentially reducing the need for more invasive procedures like surgical myectomy or heart transplantation. Larger, multicentre studies with longer follow-up periods are needed to confirm the long-term safety and efficacy of PIMSRA in this patient population.

## Introduction

 Non-obstructive hypertrophic cardiomyopathy (NOHCM) is a subtype of hypertrophic cardiomyopathy (HCM) and accounts for one-third of HCMs.^1 2^ It manifests as left ventricular outflow tract gradients (LVOTG) <30 mm Hg both at rest and with provocation.^3^ The traditional view is that LVOT obstruction is an important cause of adverse clinical events in HCM, whereas patients with NOHCM have a more optimistic clinical progression.^4^ However, approximately 15% of NOHCM cohorts are assessed to have drug-refractory symptoms without effective invasive treatment options^5^ and may enter the heart transplant queue. The time window for heart transplantation is narrow, and there exists a 10–23% chance of death while waiting for transplantation.^6^ Contributing to the poor prognosis of patients with NOHCM may be related to the microvascular dysfunction caused by myocardial hypertrophy, which further impairs myocardial diastolic and systolic functions and causes myocardial fibrosis.^7^,^9^ Therefore, we may improve clinical symptoms and functional status of patients with NOHCM by reducing the septal thickness.

In response to the clinical treatment needs of patients with NOHCM, we performed percutaneous intramyocardial septal radiofrequency ablation (PIMSRA, Liwen procedure), an original interventional ablation procedure guided by echocardiography,^10 11^ for the first time to treat patients with drug-refractory NOHCM. This study aimed to preliminarily evaluate the safety and effectiveness of PIMSRA in patients with drug-refractory NOHCM.

## Methods

### Patient population

This was a prospective exploratory study. We enrolled 20 adult patients with drug-refractory NOHCM at Xijing Hospital in Xi’an, China, between June 2017 and June 2023. All patients underwent PIMSRA after completing preoperative examination, and follow-up was performed at 1 month and 6 months after the procedure. All patients had their last follow-up before the study’s termination time (30 June 2023). Patients were aged between 19 and 74 years. All patients had severe clinical symptoms (New York Heart Association (NYHA) class II or III) and significant decreases in activity tolerance and quality of life (Kansas City Cardiomyopathy Questionnaire-12 (KCCQ-12) ≤80) despite receiving maximally tolerated medical therapy.^12^ Meanwhile, the wall thickness ≥28 mm in any region of the left ventricle by echocardiography or cardiac magnetic resonance (CMR) imaging was an inclusion criterion.^13^ Minors and patients with other cardiac conditions were excluded. All patients gave informed consent to receive PIMSRA after multidisciplinary discussion and were registered at the Chinese Clinical Trial Registry (ChiCTR1900020530). All patients underwent preoperative genetic testing for HCM.

### Echocardiography

Transthoracic echocardiography (TTE) was performed using the EPIQ 7C ultrasound system (Philips Medical Systems) with S5-1 and X5-1 transducers (1.0–5.0 MHz). ECG was acquired simultaneously. Measurements were obtained according to the recommendations of the American Society of Echocardiography.^14^ Left atrial volume is more relevant to the prognosis of patients with cardiomyopathy than left atrial anteroposterior dimension for assessment of myocardial disease.^15^

Patients underwent preoperative and postoperative myocardial contrast echocardiography (MCE). The contrast agent used was sulfur hexafluoride microbubbles (SonoVue; Bracco, 2 mL). Echocardiographic images of the different views were recorded while slowly injecting contrast agent into the peripheral vein (usually the radial vein).

We obtained three-dimensional speckle tracking echocardiography (3D-STE) strain by using TomTec software (4D LV-Analysis V.3.0; TomTec Imaging Systems, Munich, Germany). The 3D strain measurements included global radial strain (GRS), global circumferential strain (GCS) and global longitudinal strain (GLS). GRS, GCS and GLS were averaged over 16 segments. All strain values are expressed as percentages.

### ECG and ambulatory blood pressure monitoring

All patients routinely underwent 12-lead ECG, 24-hour Holter and 24-hour ambulatory blood pressure monitoring before and after PIMSRA.^16^

### Cardiac magnetic resonance

CMR scans were performed before operation using a 1.5 T magnet (Magnetom Aera, Siemens Medical Solutions, Erlangen, Germany). Each scan included balanced steady-state free precession cine images (four, two and three-chamber views). The scan included shortened Modified Look-Locker Inversion recovery sequence before and 15 min after 0.1 mmoL/kg of contrast (Magnevist, Schering, Berlin, Germany) and two-dimensional gradient echo inversion recovery for late gadolinium enhancement (LGE) images 8–10 min after contrast.

CMR images were analysed using professional image analysis software (cvi42 version 5.11). LGE was quantified in grams and per cent of left ventricular mass (LVM) using the SD method with a ≥6 SD setting. The endocardial and epicardial contours were manually traced at end-diastolic and end-systolic phases to calculate the left ventricular end-diastolic volume, left ventricular end-systolic volume, left ventricular ejection fraction (LVEF) and left ventricular mass index (LVMI) by dividing the LVM by the body surface area.

### KCCQ-12 questionnaire

Patient health status at follow-up was measured by KCCQ-12, a shortened version of the original KCCQ-23 that retains the high repeat-test reliability of the original KCCQ-23.^17^ It quantifies the following four domains: symptom frequency, physical limitations, social limitations and quality of life. Scores range from 0 to 100; the higher the score, the better the health status.^18^

### PIMSRA procedure

**Figure 1:**
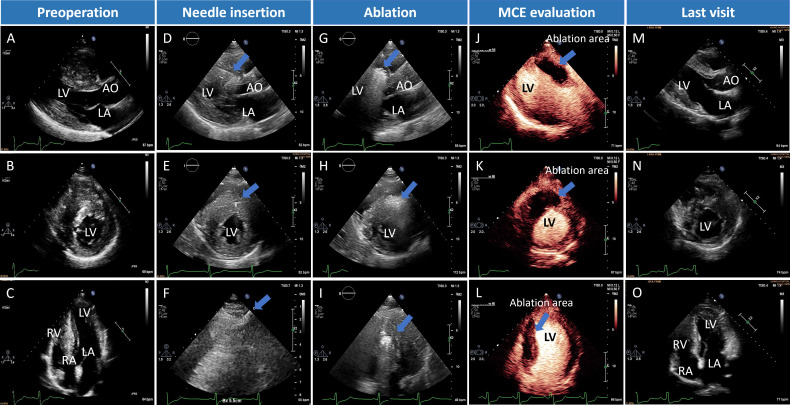
Echo imaging of percutaneous intramyocardial septal radiofrequency ablation (PIMSRA) in non-obstructive hypertrophic cardiomyopathy (NOHCM). (A–C) Preoperative echocardiography. (D–F) Intraoperative needle insertion echocardiography; arrows show the location of the radiofrequency ablation needles. (G–I) Echocardiographic images during ablation (hyperechoic zone). (J–L) Contrast echocardiography confirmed the perfusion defects in the ablated regions in the interventricular septum (IVS) immediately after the procedure. (M–O) Postoperative echocardiography. AO, aorta; LA, left atrium; LV, left ventricle; MCE, myocardial contrast echocardiography; RA, right atrium; RV, right ventricle.

The patients were in the left lateral position after general anaesthesia as previously described.^11^ Continuous ECG, blood pressure and oxygen saturation monitoring were performed. Under the real-time guidance of transthoracic echo, the radiofrequency electrode needle was inserted percutaneously from the apex through the myocardium into the hypertrophied interventricular septum (IVS). Intraoperative colour Doppler TTE guidance was used to avoid vascular injury and lungs at the point of needle placement (figure 1D–F). Using high-frequency alternating current from the tip of the radiofrequency needle, ions in the cardiomyocytes were energised to generate heat. The tissue around the radiofrequency electrode needle dehydrates, resulting in irreversible coagulative necrosis of the tissue, along with local vascular coagulation, which reduced the blood supply to the hypertrophied myocardium (figure 1G–I). If heart block or tachyarrhythmia occurred, the ablation procedure was temporarily suspended until normal rhythm was restored spontaneously or after lidocaine treatment. The radiofrequency energy was started at a low energy level (10 W) and increased slowly to avoid arrhythmias. When changing the needle path, we withdrew the tip of the needle to the apex and then gently switched the path to prevent tissue injury and pericardial effusion. The ablation time and energy delivery were adjusted according to the patient’s clinical parameters.

### Statistical analysis

Statistical analysis was performed with the SPSS V.26.0 (SPSS). Normal distribution was assessed with a normal Q-Q plot and Shapiro-Wilk test. Continuous normal distribution variables were presented as mean (SD). Continuous non-normal distributed variables were presented as median (IQR). Nominal variables were recorded using frequency (percentages). The mixed‐effects model was used for the comparison of continuous variables between baseline, 1 month and the last visit after operation. The categorical variables were compared using the McNemar’s χ^2^ test. All statistical tests were two sided, and p<0.05 was defined as statistically significant.

## Results

### Baseline characteristics

The first three patients with drug-refractory NOHCM successfully underwent PIMSRA after being approved by the Institutional Ethics Committee of Xijing Hospital. No major clinical events occurred postoperatively. Therefore, we registered our trial for this prospective study. A total of 20 adult patients with NOHCM with drug-refractory symptoms from 2017 to 2023 were enrolled in this study. Their baseline characteristics are shown in table 1. The mean age of the patients participating in the study was 43.2 (14.9) years. 11 (55%) of patients were in NYHA class II and nine (45%) were in NYHA class III. The mean preoperative N-terminal pro-B-type natriuretic peptide was 1933.8 (1091.7) pg/mL. 15 patients had palpitations, and two of them were found to have atrial fibrillation (AF) by 24-hour Holter. 11 (55%) patients had a family history of HCM. CMR suggested the presence of myocardial fibrosis in 19 patients, with a mean percentage of LGE extent of 16.2 (9.2)%. Five patients experienced syncope and presyncope despite optimal treatment.

**Table 1:** Baseline clinical characteristics

Female, n (%)	13 (65.0)
Age, mean (SD), year	43.2 (14.9)
BSA, mean (SD), m^2^	1.8 (0.2)
NYHA classification, n (%)	
Ⅱ	11 (55.0)
Ⅲ	9 (45.0)
Comorbidities, n (%)	
Hypertension	5 (25.0)
Diabetes mellitus	1 (5.0)
Coronary heart disease	0 (0.0)
Risk factors	
NT-proBNP, mean (SD), pg/mL	1933.8 (1091.7)
AF, n (%)	2 (10.0)
NSVT, n (%)	3 (15.0)
Unexplained syncope and presyncope, n (%)	5 (25.0)
LGE present, n (%)	19 (95.0)
LGE extent, mean (SD), %	16.2 (9.2)
Family history of HCM, n (%)	11 (55.0)
Medication, n (%)	
β-blocker	18 (90.0)
Calcium channel blocker	1 (5.0)
Diuretic	2 (10.0）
Genetic positive, n (%)	15 (75.0)
MYH7	6 (30.0)
MYBP3	7 (35.0)
TNNI3	1 (5.0)
TNNT2	2 (10.0)

Data are mean (SD), n (%).

AF, atrial fibrillation; BSA, body surface area; HCM, hypertrophic cardiomyopathy; LGE, late gadolinium enhancement; NSVT, non-sustained ventricular tachycardia; NT-proBNP, N-terminal pro-B-type natriuretic peptide; NYHA, New York Heart Association.

### Procedure characteristics and clinical events

Procedure data and clinical events within 30 days after PIMSRA are shown in table 2. The total radiofrequency ablation time was 73.5 (30.4) min and mean ablation power was 72.3 (22.3) W. Immediate postoperative MCE showed the length of ablation area at 42.5 (8.3) mm; the width 38.8 (9.0) mm; and the thickness 16.2 (4.8) mm. There were no major adverse clinical events such as sudden cardiac death (SCD), ventricular fibrillation or permanent pacemaker implantation within 30 days after PIMSRA. Three patients presented with non-sustained ventricular tachycardia (NSVT), none of which caused haemodynamic compromise. Two patients had complete right bundle branch block (RBBB) postoperatively. No other adverse events such as pericardial tamponade and ventricular septal defect occurred postoperatively.

**Table 2:** Mean procedure data and clinical events

Characteristic	
Mean procedure data, mean (SD)	
Total energy, kJ	309.6 (126.2)
Total ablation time, min	73.5 (30.4)
Maximum power, W	96.0 (29.1)
Mean power, W	72.3 (22.3)
Ablation area, mean (SD)	
Length, mm	42.5 (8.3)
Width, mm	38.8 (9.0)
Thickness, mm	16.2 (4.8)
Major adverse clinical events (within 30 days), n (%)	
Sudden cardiac death	0 (0.0)
VF	0 (0.0)
Permanent pacemaker implantation	0 (0.0)
Other adverse clinical events (within 30 days), n (%)	
NSVT	3 (15.0)
AF	0 (0.0)
Right bundle branch block	2 (10.0)
Left bundle branch block	0 (0.0)
Severe atrioventricular block	0 (0.0)
Pericardial tamponade	0 (0.0)
Ventricular septal defect	0 (0.0)

Data are mean (SD), n (%).

AF, atrial fibrillation; NSVT, non-sustained ventricular tachycardia; VF, ventricular fibrillation.

### Postoperative characteristics

**Figure 2:**
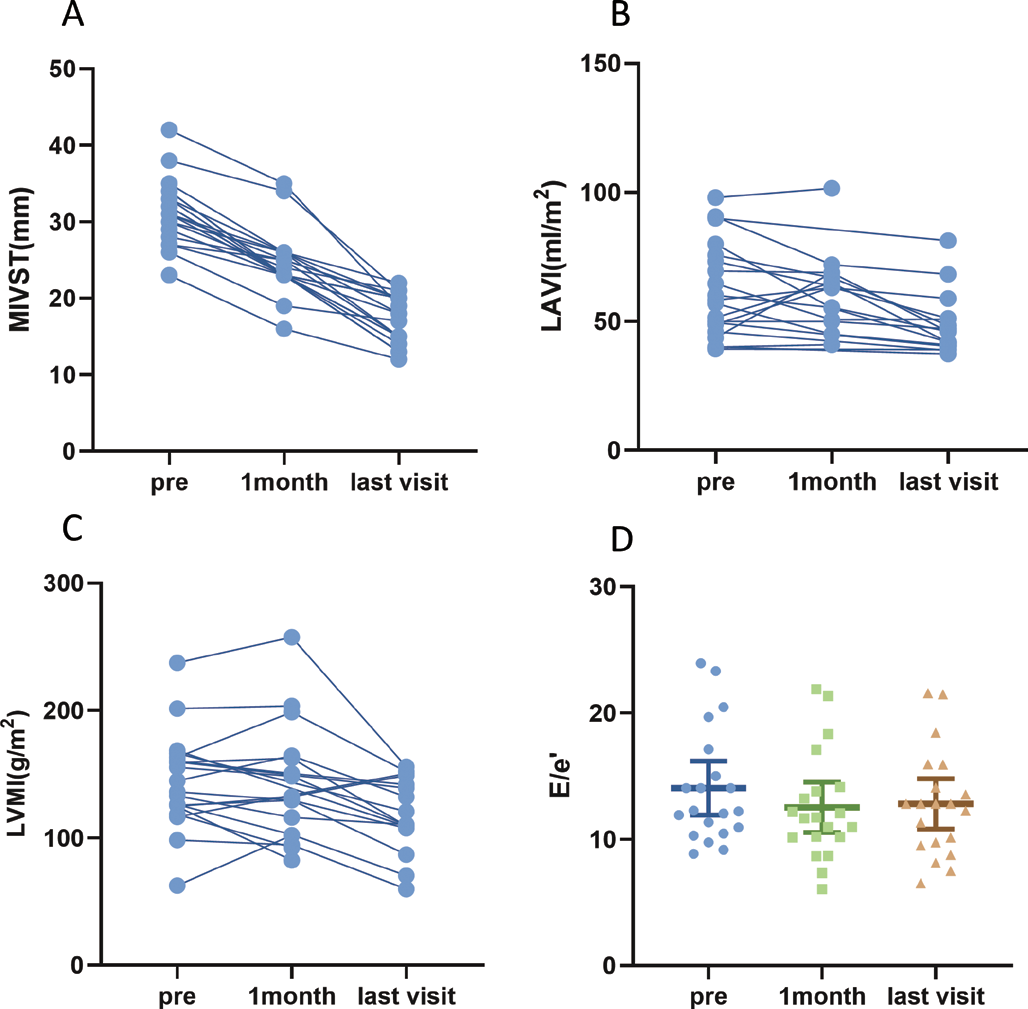
Patient-level outcomes for percutaneous intramyocardial septal radiofrequency ablation (PIMSRA) procedure. (**A**) Maximum interventricular septal thickness (MIVST) during the follow-up period. (**B**) The left atrial volume index (LAVI) during the follow-up period. (**C**) The left ventricular mass index (LVMI) during the follow-up period. (**D**) The E/e’ (mean with 95% CI) during the follow-up period.

During the median follow-up period of 15 (10, 36) months, no patient had died, no patient had developed left ventricular aneurysm or rupture and no patient developed AF or atrioventricular block. We statistically analysed the data from the patients at 1 month, 6 months postoperatively and the last visit follow-up (online supplemental table 1). PIMSRA was associated with the improvement of clinical symptoms. KCCQ-12 from 16 patients indicated clinically significant improvement in health status. The summary scores were increased from 65.6 (15.7) to 84.4 (13.3) (mean difference: 18.78; 95% CI 11.62 to 25.93), with a statistically significant improvement in physical limitation (mean difference: 16.15; 95% CI 8.46 to 25.83), symptom frequency (mean difference: 13.15; 95% CI 3.64 to 22.66) and quality of life (mean difference: 35.16; 95% CI 22.47 to 47.85). The maximum interventricular septal thickness of patients exhibited a significant reduction (31.3 (4.3) mm vs 17.4 (3.1) mm; mean difference: −13.9 mm; 95% CI −15.92 to −11.88) (table 3). During the follow-up period, a notable decrease in the left atrial volume index (LAVI) was observed from preoperative to the last postoperative follow-up (34.9 (9.8) mL/m^2^ vs 27.1 (6.0) mL/m^2^; mean difference: −7.82 mL/m^2^; 95% CI −11.50 to −4.13), and LVMI showed a statistically significant decrease from the preoperative to the last postoperative follow-up (129.2 (45.9) g/m^2^ vs 116.2 (43.8) g/m^2^; mean difference: −28.24 g/m^2^; 95% CI −18.32 to −6.44) (figure 2A–C). There is no significant change in LVEF (p=0.424).

**Table 3:** Clinical indicators of PIMSRA at 1 month and the last follow-up

	Preoperative	1 month after PIMSRA	6 months after PIMSRA	Last follow-up	Mean difference (95% CI)	P value
**Clinical characteristic**						
NYHA classification, n (%)						
I	0 (0.0）	–	10 (52.6)	10 (50.0）	–	
II	11 (55.0）	–	8 (42.1)	9 (45.0）	–	
III	9 (45.0）	–	1 (5.3)	1 (5.0）	–	
IV	0 (0.0）	–	0 (0.0)	0 (0.0）	–	<0.001
KCCQ-12, mean (SD)	65.6 (15.7)	–	85.9 (11.5)	84.4 (13.3)	18.78 (11.62 to 25.93)	<0.001
Physical limitation	71.9 (13.2)	–	86.9 (9.6)	88.0 (11.8)	16.15 (8.46 to 25.83)	<0.001
Symptom frequency	73.3 (22.2)	–	88.1 (13.8)	86.5 (20.3)	13.15 (3.64 to 22.66)	0.003
Quality of life	41.4 (17.5)	–	78.6 (18.6)	76.6 (20.3)	35.16 (22.47 to 47.85)	0.013
Social limitations	77.1 (19.4)	–	89.9 (10.4)	86.5 (12.0)	9.38 (−1.64 to 20.39)	0.258
6 min walking test, mean (SD), m	414.3 (54.2)	–	410.5 (50.0)	427.1 (60.4)	12.73 (−15.22 to 40.68)	0.196
**Echocardiography parameters**						
MIVST, mean (SD), mm	31.3 (4.3)	24.6 (4.2)	18.5 (3.7)	17.4 (3.1)	−13.90 (−15.92 to −11.88)	<0.001
LAVI, mean (SD), mL/m^2^	34.9 (9.8)	34.1 (7.1)	28.3 (6.9)	27.1 (6.0)	−7.82 (−11.50 to −4.13)	0.005
E/A, mean (SD)	1.2 (0.5)	1.5 (0.4)	1.4 (0.4)	1.3 (0.5)	0.04 (−0.19 to 0.27)	0.666
E/e’, mean (SD)	14.1 (4.6)	12.5 (4.3)	8.5 (6.9)	12.8 (4.2)	−1.24 (−3.74 to 1.25)	0.580
e’, mean (SD)	4.3 (1.5)	5.4 (1.6)	4.8 (1.3)	4.7 (1.4)	0.43 (−0.33 to 1.19)	0.712
Mitral valve SAM positive, n (%)	3 (15.0)	0 (0.0)	0 (0.0)	0 (0.0)	–	0.100
**CMR indicators**						
LVEDV, mean (SD), mL	141.6 (21.7)	–	–	141.8 (23.5)	−4.25 (−19.74 to 11.24)	0.476
LVESV, mean (SD), mL	61.8 (10.4)	–	–	64.8 (9.3)	−1.63 (−10.02 to 6.77)	0.247
LVEF, mean (SD), %	56.7 (6.0)	–	–	54.0 (4.5)	−2.28 (−6.27 to 1.71)	0.424
LVMI, mean (SD), g/m^2^	129.2 (45.9)	–	–	116.2 (43.8)	−28.24 (−18.32 to −6.44)	<0.001
**Myocardial mechanics parameters, mean (SD)**						
GLS, %	−16.6 (3.8)	−20.9 (6.0)	–	−21.8 (4.2)	−5.18 (−7.73 to −2.63)	0.003
GRS, %	32.5 (5.0)	37.3 (5.9)	–	38.1 (5.7)	5.65 (2.47 to 8.84)	0.041
GCS, %	−24.1 (3.8)	−24.7 (2.2)	–	−25.2 (4.8)	−1.11 (−3.45 to 1.24)	0.561

Data are mean (SD), n (%).

Mean differences are between the last follow-up and preoperation.

Follow-up time, median (range), month: 15 (10, 36).

CMR, cardiac magnetic resonance; GCS, global circumferential strain; GLS, global longitudinal strain; GRS, global radial strain; KCCQ-12, Kansas City Cardiomyopathy Questionnaire-12; LAVI, left atrial volume index; LVEDV, left ventricular end-diastolic volume; LVEF, left ventricular ejection fraction; LVESV, left ventricular end-systolic volume; LVMI, left ventricular mass index; MIVST, maximum interventricular septal thickness; NYHA, New York Heart Association; PIMSRA, percutaneous intramyocardial septal radiofrequency ablation; SAM, systolic anterior motion.

**Figure 3:**
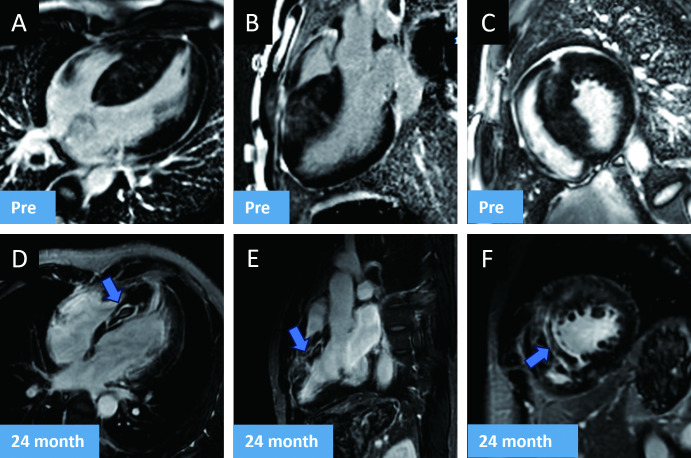
Cardiac magnetic resonance (CMR) outcome of patients after percutaneous intramyocardial septal radiofrequency ablation (PIMSRA) procedure. (A–C) Preoperative CMR. (D–F) 24-month postoperative CMR. The zone of ablation shows ongoing fibrosis and shrinkage of the ablation-induced septal lesion.

The increase in the GLS (p=0.003) and the increase in GRS (p=0.041) were statistically significant (figure 2D). Preoperative and postoperative CMR images show continuous thinning of the ventricular septum and shrinkage of the ablation-induced septal lesion (figure 3).

Patients after PIMSRA did not show an increased risk of arrhythmias such as AF and NSVT (table 4).

**Table 4:** ECG and blood pressure outcome of PIMSRA at 1 month and the last follow-up

	Preoperative	1 month after PIMSRA	6 months after PIMSRA	Last follow-up	Mean difference (95% CI)/median difference (IQR)	P value
ECG results						
Average HR, median (IQR), bpm	66.5 (12.2)	67.0 (14.0)	66.0 (13.0)	70.0 (4.8)	0.00 (−6.25, 6.75)	0.968
Single atrial premature beats, median (IQR)	5.0 (20.0)	8.0 (35.0)	5.0 (22.0)	3.5 (10.5)	0.00 (−5.38, 3.00)	0.451
Paired atrial premature beats, median (IQR)	0.0 (1.0)	0.0 (3.3)	0.0 (1.0)	0.0 (0.0)	0.00 (−0.75, 0.00)	0.282
Atrial tachycardia, median (IQR)	0.0 (0.7)	0.0 (0.0)	0.0 (1.0)	0.0 (0.0)	0.00 (0.00, 0.00)	0.390
Single ventricular premature beats, median (IQR)	6.0 (62.8)	0.0 (100.5)	1.0 (6.0)	2.5 (91.0)	−0.50 (−36.88, 15.5)	0.595
Paired ventricular premature beats, median (IQR)	0.0 (1.0)	0.0 (0.0)	0.0 (0.0)	0.0 (0.0)	0.00 (−1.00, 0.00)	0.886
VT, median (IQR)	0.0 (0.0)	0.0 (0.0)	0.0 (0.0)	0.0 (0.0)	0.00 (0.00, 0.00)	–
Blood pressure						
Mean systolic blood pressure, mean (SD)	117.1 (12.5)	114.4 (13.9)	115.0 (16.3)	120.1 (14.8)	2.94 (−1.31 to 7.20)	0.028
Mean diastolic blood pressure, mean (SD)	67.1 (12.4)	67.6 (9.0)	66.7 (9.0)	70.5 (10.6)	3.47 (−2.61 to 9.56)	0.039
SBPV, mean (SD)	7.2 (3.6)	7.2 (4.6)	7.1 (9.1)	10.0 (4.2)	2.83 (0.04 to 5.62)	0.170
DBPV, mean (SD)	10.6 (5.1)	7.2 (3.8)	9.8 (10.3)	12.8 (5.6)	1.96 (−1.67 to 5.59)	0.079

Data are mean (SD), n (%) or median (IQR), unless otherwise indicated. Mean/median differences are between the last follow-up and preoperation.

Follow-up time, median (range), month: 15 (10, 36).

bpm, beats per minute; DBPV, diastolic blood pressure variability; HR, heart rate; PIMSRA, percutaneous intramyocardial septal radiofrequency ablation; SBPV, systolic blood pressure variability; VT, ventricular tachycardia.

## Discussion

This study preliminarily explored the safety and effectiveness of drug-refractory NOHCM treated with PIMSRA. Compared with patients’ baseline status, we saw improved clinical symptoms and myocardial function, thinned IVS and no serious complications.

The traditional thinking was that the clinical progression of patients with NOHCM was relatively benign compared with obstructive HCM,^19 20^ but recent studies had indicated that long-term mortality in patients with NOHCM was underestimated.^2^ In particular, 10–15% of patients with NOHCM still have drug-refractory clinical symptoms despite guideline-recommended standardised pharmacotherapy, which greatly affects the quality of life of patients.^5 21^ However, surgical options available to these patients are very limited, possible transapical myotomy in patients with extensive apical hypertrophy for symptom relief.^3^ But this approach has been found to have a high probability of adverse clinical events, such as ventricular arrhythmias,^22^ highlighting the great unmet need for effective therapy.

The safety and effectiveness of the innovative procedure PIMSRA was validated when applied to patients with obstructive HCM.^10 11^ PIMSRA can provide precise ablation of hypertrophied myocardium and related septal branches. Therefore, PIMSRA may provide a new therapeutic option for patients with NOHCM.

With multidisciplinary team discussions, extensive review and deliberation, and final approval by our institution’s ethics committee, we treated 20 adult patients with NOHCM by PIMSRA (figure 1). The CMR results of these patients suggested that they had severe myocardial fibrosis although they did not have LVOTG (table 1). During the perioperative period, no major clinical events occurred in patients. Two patients developed complete RBBB in the postoperative period, which was considered to be related to the ablation area close to the endocardium of the right ventricle during the ablation process. It prompted us to control the ablation area more carefully in the subsequent procedure.

PIMSRA was mainly applied to the mid-layer of myocardium and did not damage the high density of longitudinally oriented myocardial fibres in the endocardium. With the ablation of hypertrophied myocardium and reduction of myocardial loading after the procedure, we observed a significant improvement in GLS during follow-up. Abnormal GLS is associated with an adverse clinical risk in patients with HCM,^23^ and improvement in GLS was an encouraging finding.^24^ Meanwhile, the sustained decrease in LAVI and LVMI demonstrated signs of improved left ventricular remodelling after ablation.^25^ However, this study observed that the preoperative LAVI and GLS of patients were not in a severe state, probably due to the absence of LVOT obstruction. Due to very severe IVS hypertrophy, we decided to perform PIMSRA after multidisciplinary consultation.

In patients with HCM and septal thickness ≥28 mm, current clinical guidelines recommend consideration of cardiac defibrillator implantation due to increased risk of SCD.^26 27^ We have not observed any obvious clinical parameters such as syncope or ventricular arrhythmia in this treated group up to this point.

Throughout the entire PIMSRA procedure, we have two strategies to protect the conduction tracts. First, we inserted transmyocardial needles into the mid-wall of the septum. The ablation process was monitored by the TTE to control the extent of ablation and to keep distance from the endocardium. Second, trained staff are responsible for monitoring the 12-lead ECG, which allows immediate detection of any abnormal conduction pattern during the ablation process. If any arrhythmia occurs, early device warnings will temporarily suspend the ablation and allow us to implement the necessary adjustments. These ablation strategies maximise the protection of the patients’ conduction system, which minimise the incidence of postoperative bundle branch block.^11^ The protection of the patients’ conduction system can be the advantage of PIMSRA in NOHCM.

### Study limitations

It is a preliminary single-centre, single-arm clinical study with the primary objective of evaluating the safety and effectiveness of PIMSRA in the treatment of only 20 patients with NOHCM, so the findings cannot be generalised to other populations. It will be important to follow patients for more than 5 years. The enrolment and follow-up need to be continued.

It is necessary to include patients with a more severe type of NOHCM to further explore the safety and effectiveness of PIMSRA. Meanwhile, there were many data points in a small patient group, so there may be some seemingly significant results purely by chance.

## Conclusion

We have preliminarily demonstrated that PIMSRA (Liwen procedure) was a safe treatment option for myocardial septal reduction in patients with NOHCM and severe myocardial hypertrophy ≥28 mm. It showed significant improvement in patients’ quality of life, haemodynamics and myocardial mechanics.

## Supplementary material

10.1136/heartjnl-2024-325334 online supplemental file 2

## Data Availability

No data are available.
