# Supplementary material for: Percutaneous intramyocardial septal radiofrequency ablation: a novel treatment for drug-refractory non-obstructive hypertrophic cardiomyopathy with severe septal hypertrophy
Source: Heart. 2025 Mar 7;111(19):e325334. doi: 10.1136/heartjnl-2024-325334 (PMC12505089; doi:10.1136/heartjnl-2024-325334)
Supplement: online supplemental file 2 [file heartjnl-111-19-s001.docx]

Supplementary table1 Patients number of follow-up tests

| Follow-up time | patient numbers | Basic information | TTE | KCCQ-12 | ECG | BPM | 6-min walk test | CMR |
| --- | --- | --- | --- | --- | --- | --- | --- | --- |
| Preoperation | 20 | 20 | 20 | 16 | 20 | 17 | 18 | 19 |
| 1 month after PIMSRA | 19 | 19 | 19 | - | 19 | 18 | - | - |
| 6 month after PIMSRA | 19 | 19 | 19 | 14 | 17 | 13 | 14 | - |
| The last follow-up | 20 | 20 | 20 | 16 | 20 | 17 | 17 | 10 |

*TTE: Transthoracic echocardiography. KCCQ-12: Kansas City Cardiomyopathy Questionnaire-12. ECG: Electrocardiogram. BPM: ambulatory blood pressure monitoring
